# Clinical characterization, molecular and genomic sequencing analysis of SARS-Cov-2 during second wave at Raigarh, Chhattisgarh, India

**DOI:** 10.6026/9732063002001059

**Published:** 2024-09-30

**Authors:** Anubha Patel, Anuniti Mathias, Ashish Baghel, Ankita Kumari, Swati Kujur

**Affiliations:** 1 Department of Microbiology, Government Medical College, Mahasamund, Chhattisgarh, India; 2 Department of Microbiology, BRLSABVM Medical College, Rajnandgaon, Chhattisgarh, India; 3 Department of Community Medicine, Government Medical College, Mahasamund, Chhattisgarh, India; 4 Department of Microbiology, LSLAMG Medical College, Raigarh, Chhattisgarh, India; 5 Scientific Officer (Biology), State Forensic Science Laboratory, Raipur, Chhattisgarh, India

**Keywords:** SARS-Cov-2, second wave, COVID -19 Molecular analysis, Genomic sequencing

## Abstract

The severe acute respiratory syndrome coronavirus-2 (SARS-Cov-2) has been changing continuously. This study was conducted to evaluate clinical characteristics, Molecular analysis & Genomic sequencing of SARS-Cov-2 during second wave in Raigarh district, Chhattisgarh, India. This study evaluated 13402 breakthrough cases of COVID -19. The laboratory obtained the nasopharyngeal/oropharyngeal swabs (NPS/OPS) of SARS-CoV-2 patients who tested positive by real-time RT-PCR, together with clinical and demographic information. Next generation sequencing (NGS) was used to sequence these clinical specimens in order to identify nucleotide changes in the SARS-CoV-2 genome from these strains. In the study population, variants of concern (VOCs) and other variations were looked for. Clinical severity was mild in 47.05% patients with mutational variants; while 52.94% patient's clinical severity was moderate. Delta (B.1.617.2) was the most common VOC detected. Among non VOC variants, AY.4 and AY.12 variants were most commonly detected. Envelope (E) gene and RNA-dependent RNA polymerase (RdRp) mutation were most commonly observed.

## Background:

India, the nation most severely impacted after the USA, handled the first wave of the COVID-19 epidemic quite successfully, but sadly suffered greatly during the second wave [1, 2- 3]. The second wave, which almost completely destroyed the country's healthcare infrastructure and caused an unheard-of increase in COVID cases and fatalities, was essentially uncontrollable and unmanaged [4, 5-6]. The conventional method for the diagnosis of COVID-19 is the identification of viral nucleic acid [2-4]. Based on the viral nucleic acid present in respiratory specimens, there are numerous molecular methods available for the identification of severe acute respiratory syndrome coronavirus-2 (SARS-CoV-2) [3-5]. The "gold standard" for verifying the diagnosis in clinical instances of COVID-19 is real-time RT-PCR on nasopharyngeal along with oropharyngeal swabs [4-7]. This method uses one or more primer-probe pairs to target SARS-CoV-2 sequences. The primer-probe sets are designed to target distinct regions of SARS-CoV-2, such as RNA-dependent RNA polymerase (RdRp) sequences, spike (S), nucleocapsid (N), envelope (E) and orf1 (a, b) [5- 7]. The RT-PCR is capable of detecting and verifying SARS-CoV-2 globally, and each gene exhibits a unique combination of sensitivity and specificity [8, 9- 10].

In December 2019, Wuhan reported the first cases of SARS-CoV-2, which quickly spread throughout the world. On March 11, 2020, the World Health Organization (WHO) deemed it an Emergency in Public Health of Worldwide Concern [11, 12-13]. Subsequently, the virus has been changing constantly. The first significant mutation was discovered in the spike-protein (D614G), which boosted the virus's contagiousness [14, 15-16]. But from September to December 2020, reports of multiple novel SARS-CoV-2 variants of concern (VOC)- Gamma (B.1.1.28.1), Beta (B.1.35) and Alpha (B.1.1.7) were obtained from Brazil, South Africa and United Kingdom respectively [17, 18, 19-20]. Due to the variations' widespread distribution, the COVID-19 pandemic was more severe, more transmissible and less protected than it was against earlier infections with the SARS-CoV-2 variant. It also responded less well to vaccinations and monoclonal antibodies [12-15]. Following the global VOC alert, foreign visitors travelling at Indian airports from various nations between December 2020 and the present were monitored and subjected to real-time reverse transcription-polymerase chain reaction (RT-PCR) specific to SARS-CoV-2 [15- 18].

VOCs, or Alpha and Beta, as well as variants of interest (VOIs), including B.1.617.3, Zeta (B.1.1.28.2), Kappa (B.1.617.1) and Eta (B.1.525) under observation, were discovered as a result of the genomic surveillance [19- 21]. An acute public health emergency has arisen in India as a result of the B.1.617 lineage's recent introduction. Further evolution of the lineage produced the sub-lineages B.1.617.1, B.1.617.2, and B.1.617.3 [20, 21, 22-23]. In the state of Maharashtra, it appears that the sub-lineage B.1.617.2 has progressively supplanted the other variations, such as Alpha VOC, B.617.3 and B.1.617.1 [24-25]. Further evolution of this variant resulted in the creation of the Delta AY.1 and Delta AY.2 strains [19-24]. This study was conducted to evaluate clinical characteristics, Molecular analysis & Genomic sequencing of SARS-Cov-2 during second wave in Raigarh district, Chhattisgarh, India.

## Methods:

This study evaluated 13402 breakthrough cases of COVID -19. Breakthrough cases are those in which SARS-CoV-2 antigen or RNA is found in a specimen taken from the respiratory system. The laboratory obtained the nasopharyngeal/oropharyngeal swabs (NPS/OPS) of SARS-CoV-2 patients who tested positive by real-time RT-PCR, together with clinical and demographic information. Next generation sequencing (NGS) was used to sequence these clinical specimens in order to identify nucleotide changes in the SARS-CoV-2 genome from these strains. In the study population, VOCs and other variations were looked for.

## Retrieval of clinical and demographic data:

Even though fully completed SRFs were sought alongwith specimens,majority of the forms submitted to the laboratory were lacking because of the heightened testing load that occurred throughout the second wave of COVID-19 in India. As a result, from May 25 to July 14, 2021, telephone interviews were performed, with each breakthrough case being called and interviewed separately. The phone interviews also assisted in completing any gaps in the data and verifying the information contained in the SRF. Questioning the patients covered demographics, vaccination history, contact history with laboratory-confirmed COVID-19 cases before the breakthrough infection, presence of co-morbidities, history of prior COVID-19 infection, course of infection, including hospitalization details and symptoms.

## RNA extraction and next generation sequencing:

Using the Magmax RNA extraction kit (Applied Biosystems, USA) and automated RNA extraction equipment (Thermofisher, USA), total RNA was isolated from 200-400 µl of NS/OS swab samples. As previously mentioned [20-24], SARS-CoV-2 specific primers were used to set up real-time RTPCR for the identification of the E and RdRP genes. The E and RdRP genes' RT-PCR Ct values were determined and assessed. RT-PCR positive samples were sent to ILS Bhubaneshwar for genomic sequencing for identification of circulating COVID-19 strains as per the government directives. The result obtained was then analysed for distribution of variants and demographic and clinical characterization.

## Statistical analysis: 

Data was entered in Microsoft excel software, was checked for its completeness, correctness & was analyzed by using SPSS 21.0 version software. Descriptive statistical analysis was carried out in the present study. Results on categorical measurements were presented in numbers (%). Chi-square tests were used to find the significance of study parameters on categorical scale between two or more groups. P-value of <0.05 was considered to be statistically significant. Statistical analysis considered sensitivity, specificity; Positive Predictive Value (PPV) and Negative Predictive Value (NPV), accuracy, Kappa coefficient, and Wilson score Confidence Interval at 95% (GraphPad Prism version 9.0.1).

## Results:

**Figure 1:**
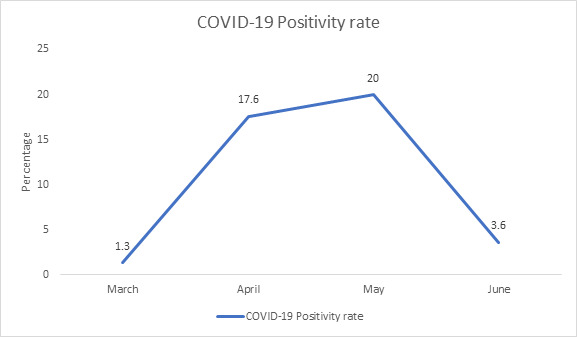
Month wise positivity rate of SARS-CoV-2-RT-PCR during COVID-19 2nd wave

The average positivity rate of samples tested in our laboratory during second wave of COVID-19 was 10.2%. The positivity rate during the wave increased from 1.3% in the month of March to 17.6% and 20% in April and May months respectively, followed by decrease to 3.6% in June [Figure 1].

In this study, 13402 study participants with COVID-19 were included. 8019 (59.83%) were male while 5383 (40.17%) were females. Males were significantly greater than females (Table 1).

Most of the study participants (64.15%) were in the age group of 18-45 years followed by 46-60 years (16.88%) as shown (Table 2).

In this study 188 cases out of 13402 cases of COVID-19 were found to have mutant variants. Overall frequency of cases with mutations was 1.40%. VOC was detected in 134 (71.2%) cases of COVID -19 with mutant variants. Variants other than VOC constituted 54 (28.73%) of total COVID -19 cases (Table 3).

Delta (B.1.617.2) was the most common VOC detected. It was detected in62.76% of COVID-19 cases with mutant variants. Some other VOCs detected were AY.46.1, AY.16.1, AY.44, AY.75, AY.9.2 and AY.39. Among non VOC variants, AY.4 and AY.12 variants were most commonly detected constituting 13.20% and 11.7% of total cases of COVID-19 with non VOCs variants. Some other non VOC variants detected were AY.5, AY.16, B.1.575, B.1.153, B.1 and AY.26.The findings were significant statistically (Table 4).

Demographic details of COVID-19 cases with mutant variants were recorded (Table 5).102 (54.25%) were not vaccinated. Covishield was the most common vaccine received. None of these cases were found to have international travel history. 77 (40.95%) were Suspected Reinfection Case. Most of patients (90.95%) underwent home isolation. Among 17 patients who got hospitalized, 47.05% patient's clinical severity was mild; while 52.94% patient's clinical severity was moderate. 2(11.76%) of patients who were hospitalized were admitted in ICU. 3 (1.60%) of patients with CIVID-19 mutant variants died while remaining were discharged.

The mean RT-PCR Ct values for E-gene and RdRP gene was 29.05±1.26 and 28.50±1.32 respectively. The overall sensitivity, specificity, PPV, NPV, accuracy, Kappa coefficient and 95%CI was higher and comparable for both E gene and RdRP gene in diagnosing COVID cases with mutational variants (Table 6).

## Discussion:

The virus SARS-Cov-2 has been changing continuously [3-7]. This study was conducted to evaluate clinical characteristics, Molecular analysis & Genomic sequencing of SARS-Cov-2 during second wave in Raigarh district, Chhattisgarh, India. In this study 188 cases out of 13402 cases of COVID-19 were found to have mutant variants. Overall frequency of cases with mutations was 1.40%. VOC was detected in 134 (71.2%) cases of COVID -19 with mutant variants. Variants other than VOC constituted 54 (28.73%) of total COVID -19 cases. Delta (B.1.617.2) was the most common VOC detected. It was detected in 62.76% of COVID-19 cases with mutant variants. Some other VOCs detected were AY.46.1, AY.16.1, AY.44, AY.75, AY.9.2 and AY.39.Among non VOC variants, AY.4 and AY.12 variants were most commonly detected constituting 13.20% and 11.7% of total cases of COVID-19 with non VOCs variants. Some other non VOC variants detected were AY.5, AY.16, B.1.575, B.1.153, B.1 and AY.26.The findings were significant statistically. The findings of present study are having resemblance with the findings of some research that also reflected a frequency of 1% to 2% of cases with mutant variants of COVID-19 in second wave of COVID -19 in India [13- 18]. The genomic monitoring led to the discovery of VOCs, or Alpha and Beta, as well as variations of interest (VOIs), such as B.1.617.3, Zeta (B.1.1.28.2), Kappa (B.1.617.1), and Eta (B.1.525) under observation [12- 21]. The emergence of the B.1.617 lineage has resulted in a serious public health emergency in India. Sub-lineages B.1.617.1, B.1.617.2, and B.1.617.3 were created by the lineages further evolution [16- 23]. It seems that the sub-lineage B.1.617.2 has gradually replaced the other varieties, including Alpha VOC, B.617.3, and B.1.617.1 [12-19]. The Delta AY.1 and Delta AY.2 strains are the product of further evolution of this variation [15- 21].

In our study, it was observed that 102 (54.25%) cases of COVID-19 with mutant variants were not vaccinated. Covishield was the most common vaccine received. None of these cases were found to have international travel history. 77 (40.95%) were suspected reinfection case. Most of patients (90.95%) underwent home isolation. Among 17 patients who got hospitalized, 47.05% patient's clinical severity was mild; while 52.94% patient's clinical severity was moderate. 2(11.76%) of patients who were hospitalized were admitted in ICU. 3 (1.60%) of patients with COVID-19 mutant variants died while remaining were discharged. Some other research carried out on different populations of COVID-19 for detection of mutant variants found results similar to results of present study [19- 25]. They also observed that proportion of patients who were not vaccinated earlier was greater as observed in our study. However, previously vaccinated individuals were also re-infected as observed in our study. This finding was observed in some other research also [21-24]. The COVID-19 pandemic second wave was more severe, more transmissible, and less protected against prior infections with the SARS-CoV-2 variant because of the variants' extensive dissemination. Additionally, it did not react as well to monoclonal antibodies or vaccines [18-23]. The spike-protein (D614G) was found to have the first notable mutation, increasing the virus's contagiousness. However, reports of several additional SARS-CoV-2 variants of concern (VOC)-Gamma (B.1.1.28.1), Beta (B.1.35), and Alpha (B.1.1.7)-were found [14-18]. In the wake of the worldwide variant of concern (VOC) alert, international travellers arriving in Indian airports, Real-time reverse transcription-polymerase chain reaction (RT-PCR) targeted at SARS-CoV-2 was conducted [11- 19].

In our study, the mean RT-PCR Ct values for E-gene and RdRP gene was 29.05±1.26 and 28.50±1.32 respectively. The overall sensitivity, specificity, PPV, NPV, accuracy, Kappa coefficient and 95%CI was higher and comparable for both E gene and RdRP gene in diagnosing COVID-19 cases with mutational variants. These findings are having similarity with the findings of some other research which like our study showed RT-PCR Ct values for E-gene and RdRP gene as 29-31 [13- 19]. Some research also showed high accuracy and sensitivity for RT-PCR targeting E-gene and RdRP gene as observed in our study [14-20]. The detection of viral nucleic acid is the standard procedure for COVID-19 diagnosis. SARS-CoV-2 can be identified using a variety of molecular techniques based on the viral nucleic acid found in respiratory specimens [15-23]. When it comes to clinical cases of COVID-19, real-time RT-PCR on nasopharyngeal and oropharyngeal swabs is the recommended approach for diagnosis verification. This technique targets SARS-CoV-2 sequences using one or more primer-probe pairs [11- 17]. The primer-probe sets are intended to target specific sections of SARS-CoV-2, including orf1 (a, b), spike (S), nucleocapsid (N), envelope (E), and RNA-dependent RNA polymerase (RdRp) sequences [16- 23]. SARS-CoV-2 can be found and confirmed worldwide with RT-PCR, and each gene has a distinct mix of sensitivity and specificity [18-25].

## Conclusion:

Clinical severity was mild in 47.05% patients with mutational variants while 52.94% patient's clinical severity was moderate. Delta (B.1.617.2) was the most common VOC detected. Among non VOC variants, AY.4 and AY.12 variants were most commonly detected. Continued genomic surveillance for identifying the emergence of any newer variants is the need of the hour for early detection and therefore, timely prevention and control of any further severe COVID-19 waves like the deadly second wave.

## Figures and Tables

**Table 1:** Distribution of study participants according to gender

	**N**	**%**	**P value**
Male	8019	59.83
Female	5383	40.17	0.012
Total	13402	100

**Table 2:** Distribution of study participants according to age

**Age group**	**N**	**%**	**P value**
Infant	34	0.25
2-5 years	205	1.52
5-10 years	412	3.07
11-17 years	1056	7.87	0.021
18-45 years	8598	64.15
46-60 years	2263	16.88
61-65 years	423	3.15
66-97years	411	3.06

**Table 3:** Details regarding mutations and variants

**Total cases evaluated**	**13402**
Cases with mutation detected	188
Frequency of cases with mutations detected (%)	1.4
VOC	134 (71.2%)
Variants other than VOC	54 (28.73%)

**Table 4:** Distribution of variants detected (n=188)

**N**	**%**
**VOC**
Delta (B.1.617.2)	118	62.76
AY.46.1	1	0.53
AY.16.1	2	1.06
AY.44	5	2.65
AY.75	4	2.12
AY.9.2	1	0.53
AY.39	1	0.53
Total	134	71.27
Variants other than VOC
AY.4	25	13.29
AY.5	2	1.06
AY.16	1	0.53
AY.12	22	11.7
B.1.575	1	0.53
B.1.153	1	0.53
B.1	1	0.53
AY.26	1	0.53
Total	54	28.73
χ^2^	0.876
df	4
P value	0.001

**Table 5:** Clinical characterization of cases detected with mutation variants (n=188)

**Vaccination received (n=188)**	**N**	**%**
Yes	68	36.2
No	102	54.3
Unknown	18	9.57
Name of vaccine received (=68)
Covishield	62	91.2
Covaxin	6	8.83
Second dose of COVID vaccine (n=68)
Received	31	45.6
Not received	37	54.4
International Travel history
Yes	0	0
No	188	100
Suspected Reinfection Case (n=188)
No	101	53.7
Yes	77	41
Management protocol (n=188)
Home isolation	171	91
Hospitalization	17	9.05
Clinical severity of hospitalized patients (n=17)
Mild	8	47.1
Moderate	9	52.9
Admission in ICU (n=17)
Yes	2	11.8
No	15	88.2
Outcome (n=188)
Discharged	185	98.4
Death	3	1.6

**Table 6:** Comparison of RT-PCR Ct values and diagnostic parameters of E gene and RdRP gene

	**E-gene**	**RdRP gene**
RT-PCR Ct values (Mean±SD)	29.05±1.26	28.50±1.32
Sensitivity	94.12	95.23
Specificity	95.23	96.14
PPV	94.17	95.41
NPV	97.19	96.28
Accuracy	98.14	96.17
Kappa coefficient	0.92	0.93
95% CI	Reference =1	1.2 (0.9-1.3)
